# Topical 5-aminolevulinic acid-mediated photodynamic therapy versus loop electrosurgical excision procedure in the treatment of cervical intraepithelial neoplasia

**DOI:** 10.1007/s44178-024-00127-3

**Published:** 2024-11-28

**Authors:** Yidi Liu, Yi Li, Huan Wu, Ying Wang, Jing Zeng, Hui Li, Haixia Qiu, Ying Gu

**Affiliations:** 1 Department of Laser Medicine, the First Medical Centre, Chinese PLA General Hospital, Beijing, 100853 China; 2 Medical School of Chinese PLA, Beijing, 100853 China; 3 Medical Big Data Center, Chinese PLA General Hospital, Beijing, 100853 China; 4 Department of Oncology, the Fifth Medical Center, Chinese PLA General Hospital, Beijing, 100071 China; 5 Precision Laser Medical Diagnosis and Treatment Innovation Unit, Chinese Academy of Medical Sciences, Beijing, 100000 China

**Keywords:** 5-aminolevulinic acid, Photodynamic therapy, Loop electrosurgical excision procedure, Cervical intraepithelial neoplasia, Squamous intraepithelial lesion, Human papillomavirus

## Abstract

**Objective:**

To compare effectiveness of topical 5-aminolevulinic acid-mediated photodynamic therapy (5-ALA PDT) and loop electrosurgical excision procedure (LEEP) among patients with cervical intraepithelial neoplasia (CIN).

**Methods:**

We retrospectively identified patients who underwent either 5-ALA PDT or LEEP from Sep. 2012 to Dec. 2019 in Chinese PLA general hospital. Patients’ outcomes were compared according to the HPV genotyping, cytological tests within 3–6-month follow-up post-treatment, the pathological examination would be performed if the cytological results indicated the risk of CIN. Propensity score matching (PSM) was adapted to pair the baseline. Complete remission (CR), partial remission (PR) and the remission rate of HPV infections were used to evaluate the efficacy of 5-ALA PDT versus LEEP.

**Results:**

In total, 30 pairs were matched as the matching tolerance was set as 0.03. There was no significant difference about the CR and PR between 5-ALA PDT and LEEP group (73.33% vs 84.00%, *P* = 0.340; 3.33% vs 4.00%, *P* = 1.000). Among different CIN group, there was no statistic difference between 5-ALA PDT and LEEP. Moreover, in terms of HPV remission rate, 5-ALA PDT showed the same efficacy as LEEP (59.26% vs 53.85%, *P* = 0.691).

**Conclusions:**

In essence, topical 5-ALA PDT emerges as a non-invasive, repeatable procedure with minimal side effects for cervical lesions, preserving cervical structure. Overall, the efficacy of 5-ALA PDT is comparable to LEEP in achieving successful outcomes.

## Introduction

Cervical Intraepithelial Neoplasia (CIN) is a precursor to invasive cervical cancer and ranks as the fourth most common malignancy among women in developing countries. Despite screening program has made advances, cervical cancer still has an attendant high fatality rate. If no intervention is implemented, based on existing epidemiological evidence in both urban and rural areas of mainland China, the annual incidence of new cervical cancer cases is predicted to significantly increase. Estimates range from approximately 27,000 to 130,000 in 2010 to reaching approximately 42,000 to 187,000 in 2050 [1]. Continued infection with the human papillomavirus (HPV) which is known as the most common sexually transmitted virus [2] causes cervical cancer and CIN. HPV genotypes have been classified into high-risk genotypes (HR-HPV) and low-risk genotypes (LR-HPV) according to the associated risk of carcinogenesis in the uterine cervix. HPV 16, 18, 31, 33, 35, 39, 45, 51, 52, 56, 58, and 59 genotypes have been identified as the HR-HPV [3]. Persistent HR-HPV infection not only increases the risk of CIN and cervical cancer, but also puts psychological burden on women with HPV infection. Therefore, patients with CIN and HR-HPV-positive tend to acquire intervention to minimize the risk of cervical lesions and prevent the occurrence of cervical malignancy.

According to how much epithelial tissue is affected, CIN can be graded on 1–3 scale, where CIN3 is the most abnormal grade. The 2014 World Health Organization Classification of female genital tumors is: (1) normal, (2) low-grade squamous intraepithelial lesion (LSIL), (3) high-grade squamous intraepithelial lesion (HSIL), and (4) squamous cell carcinoma, CIN1 is equivalent to LSIL, while CIN2, 3 are classified into HSIL. Currently, in clinical practice, surgical excision is the primary treatment for CIN, which includes using the cold-knife conization and loop electrosurgical excision procedure (LEEP). The cold-knife conization now is the standard therapy for CIN3 and early invasive cancer [4], however, it could increase the risk of premature birth. LEEP has been referred to as a feasible, tolerable method with favorable postoperative outcomes for CIN [5]. However, there are increasing number of literatures reported LEEP has a variety of complications during pregnancy including miscarriage, perinatal death, premature delivery, severe hemorrhage, incomplete excision [6]. In late 20 years, the effectiveness of photodynamic therapy with 5-aminolevulinic acid (5-ALA PDT) for CIN and HPV-infected sites have been verified [7]. 5‐ALA is absorbed by active cells selectively and converted into photoactive protoporphyrin IX (PpIX) [8]. By using laser with the specific wavelength, high levels of reactive oxygen species (ROS) are accumulated in premalignant cells to induce apoptosis and necrosis, with less damage to adherent and underlying normal cells than radiation, chemotherapy, or surgery. This new method has been shown to be effective in the treatment of CIN, which can target the lesion while preserve the normal deeper matrix and connective tissue. It provides an effective and safe alternative treatment for CIN, and avoids the risk of cervical dysfunction or scarring associated with surgical excision [9]. However, 5-ALA PDT is a relatively new method, its efficacy and whether it can be an alternative of LEEP should be investigated thoroughly.

To our knowledge, there is no report on the comparison between clinical outcomes of 5-ALA PDT and LEEP in the treatment of CIN. This article retrieved medical records of patients who received LEEP and 5-ALA PDT in Chinese PLA General Hospital within 7 years from 2012 to 2019 and we retrospectively analyzed and compared the effectiveness of these two methods by using propensity score matching (PSM).

## Materials and methods

### Study design and ethical considerations

This study was a retrospective study. All procedures performed in this study were conducted according to the ethical standards of the Institutional and National Research Committee, and the 1964 Declaration of Helsinki and its later amendments or comparable ethical standards. Informed consent was obtained from all included participants. This study was approved by the Institutional Review Board of Chinese PLA General Hospital. This retrospective analysis did not include patients’ identifiers.

### Study population

**Fig. 1:**
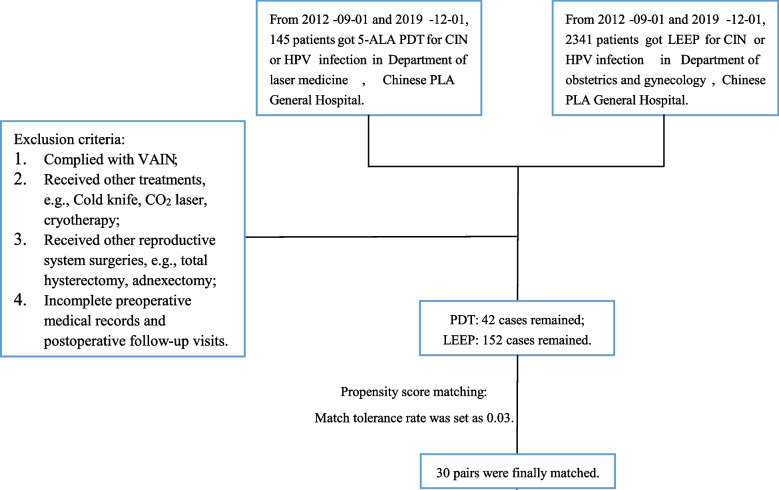
Screening flowchart. CIN, cervical intraepithelial neoplasia; HPV, human papillomavirus; VAIN, vaginal intraepithelial neoplasia

The inclusion criteria: (1) From 2012–09-01 and 2019–12-01, a total of 145 female patients with a histologically confirmed CIN or HPV infection were treated by 5-ALA PDT in the department of laser medicine of Chinese PLA General Hospital. (2) Simultaneously, A total of 2,341 patients treated by LEEP for CIN or HPV infection were selected at department of obstetrics and gynecology of Chinese PLA General Hospital (Fig. 1). Then exclusion criteria were set: (1) CIN complied vaginal intraepithelial neoplasia (VAIN); (2) Received other treatments for CIN, e.g., cold knife, CO_2_ laser, cryotherapy; (3) Received other reproductive system surgeries, e.g., total hysterectomy, adnexectomy etc.; (4) Incomplete preoperative medical records and postoperative follow-up visits. Finally, totally 42 cases and 152 cases remained for 5-ALA PDT and LEEP, respectively. To balance the baseline, propensity score matching (PSM) was adapted to pair the two groups and the match tolerance rate was set as 0.03. Thirty pairs were finally matched.

### HPV genotyping

DNA extraction and HPV genotyping were carried out using HPV genotyping real-time PCR kit (Shanghai ZJ Bio-Tech Co., Ltd) to detect the following 18 HPV types: HPV6, 11, 16, 18, 21, 31, 33, 35, 39, 45, 51, 52, 56, 58, 59, 66, 67, 68, 82. The lowest detection limit of the kit was 1 × 10^4^ copies/mL. Amplification techniques performed on SLAN®-96P (Shanghai Hongshi Medical Technology Co., Ltd) were used for the quantitative estimation of HPV DNA copies [10].

### Cytological tests

Cytological tests were performed using liquid-based cytology (ThinPrep Hologic) to evaluate abnormal cytology defined as atypical squamous cells of undetermined significance (ASCUS) or worse [10].

### Histological classification

If the cytological results indicated the risk of CIN, the pathological examination would be performed. The diagnosis of cervical cytology was classified by the 2001 Bethesda system. Experienced pathologists in the Chinese PLA General Hospital reviewed every histology slide and classified each finding as negative, CIN grade 1/2/3.

### 5-ALA Photodynamic therapy

Preparation and administration: The 5-ALA used in PDT was supplied by Shanghai Fudan-Zhangjiang Bio-Pharmaceutical (118 mg/bottle). The thermogel was also supplied as powder by Shanghai Fudan-Zhangjiang Bio-Pharmaceutical. The dosage of 5-ALA was determined by the drug delivery area, which usually to be 354 mg (3 bottle of 5-ALA). 5-ALA was dissolved in 1.5 ml thermogel at 4 ℃ following the instruction of the manufacturer. 1.5 ml of ALA-thermogel, absorbed on the gauze, in a concentration of 20% were topically applied to the cervix for 4 h. A condom, filled with medical cotton ball, was used to prevent the gauze from dropping. Patients were counseled to go to bathroom before administration and stay supine during incubation.

**Fig. 2:**
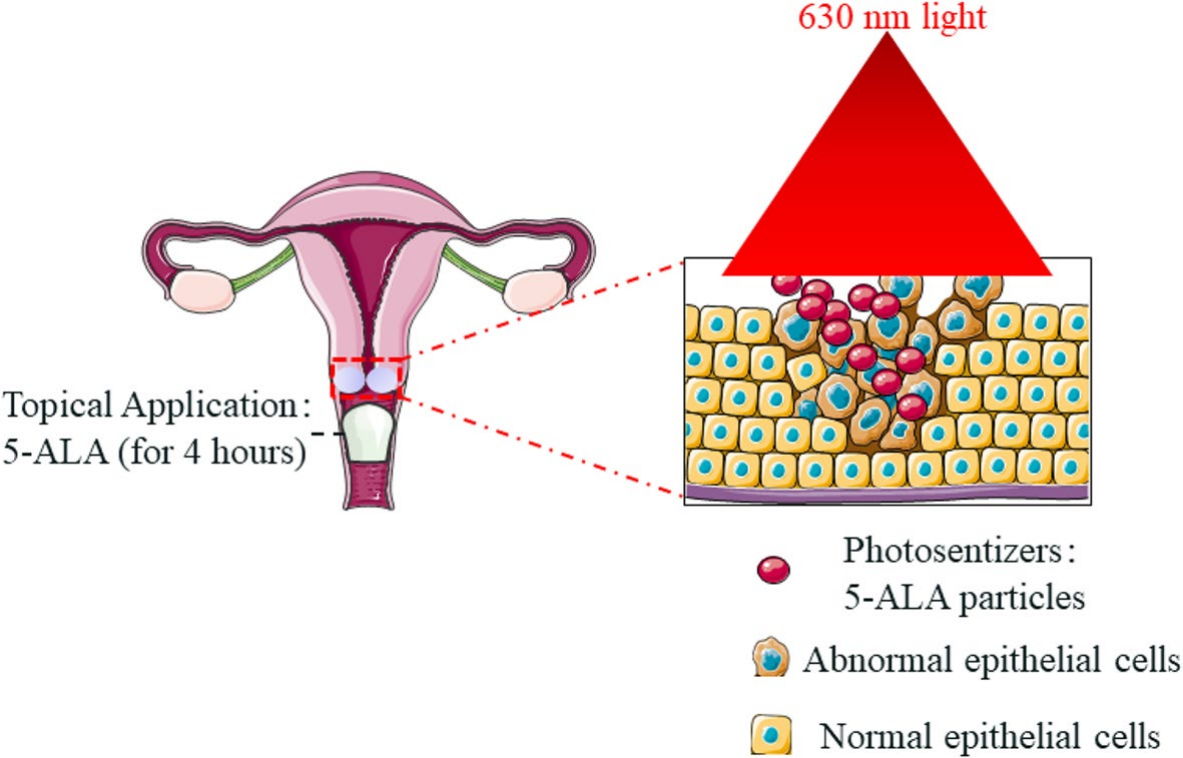
The procedure of 5-ALA photodynamic therapy

Illumination: after removal of the 5-ALA gel, the cervix was illuminated by red coherent light of a wave length of 630 nm using a lens fiber. This applicator illuminated the cervix for 20 min, administering a light dose of 120 J/cm^2^. After the intervention, the possible side effects were inquired (Fig. 2).

If the HPV genotyping or cytological tests after the treatment suggest that the lesion has not regressed, the second PDT can be adapted.

### Loop electrosurgical excision procedure (LEEP)

LEEP was performed by experienced doctor in department of obstetrics and gynecology with senior professional title according to standard practice. Hemostasis was achieved after LEEP with electrocoagulation. Then the specimens were evaluated by 2 pathologists, independently.

### Follow-up plan and effect evaluation

HPV and TCT were performed at 3 months and 6 months follow-up post-treatment, and colposcopic biopsy was performed when necessary. The effect evaluation was classified into the following aspects according to HPV, TCT and colposcopic biopsy. The primary responses were determined by the complete remission (CR) and partial remission (PR). CR was defined as the directed cytology returned to normal while PR referred to the directed cytological outcome decreased compared with the diagnosis before the treatment (e.g., CIN2-3 to CIN1). The patients who diagnosed as ASCUS by cytological tests and without pathological tests after the treatment were defined as invalid. The secondary response was the clearance rate of HPV infections, in which situation there is no evidence of positive HPV results. If the HPV genotyping or cytological tests after the treatment suggest that the lesion has not regressed, the second PDT can be adapted; (2) The interval between treatments was one month.

### Statistical analysis

In the light of the differences in the baseline characteristics between eligible participants in the two groups (Table 1), propensity-score matching (PSM) was used to identify a cohort of patients with similar baseline characteristics. The propensity score is a conditional probability of having a particular exposure (age, CIN grade, HPV infection, gravidity, production, marital status). Matching was performed with the use of a 1:1 matching protocol without replacement (greedy-matching algorithm), considering the numbers and accuracy of the matching, the match tolerance was set as 0.03. The comparison between clinical characters and outcomes were carried out by SPSS software (Version 26.0 SPSS, Inc.). A Chi-square test and Fisher’s exact tests was utilized for the counting analysis, and a t-test was used for variable data. A P value of < 0.05 was considered significant.

**Table 1:** Clinical Characteristics before propensity score matching (*n* = 194)

	ALA-PDT	LEEP	T/Z	P
Age	38.05 ± 8.65	44.16 ± 10.17	3.551	< 0.001
Diagnosis			-6.730	< 0.001
HPV-Total			-4.558	< 0.001
HPV-High risk			-3.753	< 0.001
HPV-Low risk			-2.442	0.015
Gravidity	2.33 ± 2.02	2.76 ± 1.73	1.366	0.173
Production	1.02 ± 0.60	1.38 ± 0.91	2.381	0.018
Marital Status			-0.326	0.744

## Results

### Clinical features of patients undergoing PDT or LEEP treatments

Clinical characteristics were gathered from 42 patients undergoing 5-ALA PDT and 152 undergoing LEEP (Table 1). The mean age in the 5-ALA PDT group was 38.05 ± 8.65 years, compared to 44.16 ± 10.17 in the LEEP group. Gravidity was 2.33 ± 2.02 for 5-ALA PDT and 2.76 ± 1.73 for LEEP, while parity was 1.02 ± 0.60 and 1.38 ± 0.91 for 5-ALA PDT and LEEP, respectively.

To ensure clinical outcome comparability, baseline characteristics such as age, CIN diagnosis, HPV genotyping, gravidity, parity, and marital status were compared between groups. Except for gravidity and marital status, other characteristics showed statistical differences. PSM was then applied to balance these factors, setting the matching tolerance at 0.03 for both accuracy and scale. Subsequently, 30 pairs were matched, and all clinical data, including age, diagnosis before (Dx-B) and after treatment (Dx-A), HPV infection (HPV-T), HR-HPV infection (HPV-H), gestation/parturition (G/P), marital status (MS), and number of treatments (Nx), were presented in Table 2.

**Table 2:** Clinical Characteristics after propensity score matching (*n* = 60)

Case	Tr	Age	Before	After	Hpv-T-B	Hpv-T-A	Hpv-H-B	Hpv-H-A	G/P	MS	Nx
1	0	54	1	0	1	0	0	0	4/1	1	1
2	1	23	1	0	1	0	1	0	2/0	0	1
3	0	40	1	0	1	0	1	0	3/1	1	1
4	1	46	0	1	1	0	1	0	3/2	1	1
5	0	40	1	ASCUS	1	0	1	0	2/1	1	2
6	1	44	1	0	1	0	1	0	6/1	1	1
7	0	31	1	0	1	0	1	0	5/1	1	1
8	1	30	1	0	1	1	1	1	2/1	1	1
9	0	22	2	0	1	0	1	0	0/0	0	1
10	1	41	2	0	1	0	0	0	3/1	1	1
11	0	34	1	0	1	0	1	0	0/0	0	1
12	1	30	1	0	1	0	1	0	2/1	1	1
13	0	39	1	0	1	0	1	0	2/1	1	1
14	1	37	1	1	1	1	1	1	1/1	1	1
15	0	34	1	0	1	0	1	0	1/1	1	3
16	1	33	1	1	1	1	1	1	2/1	1	1
17	0	51	1	0	1	1	1	1	1/1	1	1
18	1	39	2	0	1	0	1	0	2/1	2	1
19	0	36	1	1	1	1	1	1	1/1	1	3
20	1	33	1	1	1	1	1	1	3/2	1	1
21	0	35	1	0	1	0	0	0	2/2	1	1
22	1	28	1	1	1	1	1	1	0/0	1	1
23	0	35	1	0	1	0	1	0	3/2	1	3
24	1	35	2	1	1	1	1	1	2/2	0	1
25	0	33	1	1	0	1	0	1	0/0	2	1
26	1	44	1	0	1	0	1	0	3/2	1	1
27	0	31	2	0	1	1	1	1	0/0	0	3
28	1	48	1	0	1	0	1	0	1/0	1	1
29	0	32	1	ASCUS	0	1	0	1	6/1	1	3
30	1	48	3	0	1	1	1	1	0/0	1	1
31	0	44	1	ASCUS	1	0	0	0	4/1	1	3
32	1	28	0	0	1	0	1	0	2/1	1	1
33	0	60	1	0	0	0	0	0	0/0	1	2
34	1	53	2	0	0	0	0	0	2/1	1	1
35	0	44	1	0	1	1	1	1	3/1	1	1
36	1	31	1	0	1	0	1	0	3/2	1	1
37	0	41	1	0	1	1	1	1	2/2	1	1
38	1	28	3	0	1	1	1	1	0/0	1	1
39	0	30	1	0	1	1	1	1	0/0	0	1
40	1	44	0	0	1	0	1	0	2/1	1	1
41	0	51	1	0	1	0	1	0	1/1	1	2
42	1	31	2	0	1	0	1	0	2/1	1	1
43	0	30	2	0	1	0	1	0	2/2	1	2
44	1	43	1	0	0	1	0	1	0/0	1	1
45	0	30	3	0	1	1	1	1	1/0	0	2
46	1	47	2	0	1	1	1	1	3/1	1	1
47	0	34	1	ASCUS	1	1	1	1	2/1	1	1
48	1	41	0	0	1	1	1	1	4/2	1	1
49	0	38	1	0	1	0	1	0	7/2	1	1
50	1	38	1	0	1	0	1	0	4/2	1	1
51	0	38	1	ASCUS	1	0	1	0	3/1	2	1
52	1	51	0	0	1	0	1	0	2/1	1	1
53	0	39	2	0	1	0	1	0	2/1	1	1
54	1	55	1	0	1	1	1	0	4/2	2	1
55	0	44	2	1	1	1	1	1	2/2	1	2
56	1	30	2	0	0	0	0	0	1/1	1	1
57	0	63	1	0	1	1	1	1	1/1	1	1
58	1	23	2	0	0	0	0	0	0/0	0	1
59	0	28	2	0	1	1	1	1	2/2	1	1
60	1	44	2	0	1	0	1	0	2/1	1	1

*Abbreviations:* Tr: Treatment; 0 for 5-ALA PDT, 1 for LEEP;Before: Diagnosis before the treatment, 0 for HPV ( +), 1 for CIN1, 2 for CIN2, 3 for CIN3; HPV-T: HPV infection; 0 for no HPV infection; 1 for any type of HPV infection. HPV-H: High risk HPV infection; 0 for no high-risk HPV infection; 1 for any type of high-risk HPV infection. MS: Marital Status, 0 for unmarried, 1 for married, 2 for divorced. Nx: Number of treatments; After: Diagnosis after the treatment; ASCUS: atypical squamous cells of undetermined significance

For clear presentation, Table 3 summarized the treatment frequency and HPV infection for all 60 patients. In the 5-ALA PDT group, among the 30 patients, 23, 6, and 1 were diagnosed with CIN1, CIN2, and CIN3, respectively. Out of the 23 patients diagnosed with CIN1, 15 underwent one session, 3 had two sessions, and 5 received three sessions of PDT treatment. In the LEEP group, among the 30 patients, 14 were diagnosed with CIN1, 9 with CIN2, 2 with CIN3, and 5 as normal but with persistent HR-HPV infection; all patients received only one-time treatment.

**Table 3:** Number of treatments and HPV infection in CIN1-3 and HPV positive patients (*n* = 60)

	5-ALA PDT							LEEP					
	Nx					HPV infection rate		Nx				HPV infection rate	
	One	Two	Three	Total	Overall		High-risk	One	Two	Three	Total	Overall	High-risk
CIN1	15	3	5	23	91.30% (20/23)		78.26% (17/23)	14	0	0	14	92.86% (13/14)	92.86% (13/14)
CIN2	3	2	1	6		100% (6/6)	100% (6/6)	9	0	0	9	66.67% (6/9)	55.56 (5/9)
CIN3	0	1	0	1		100% (1/1)	100% (1/1)	2	0	0	2	100% (2/2)	100% (2/2)
HPV ( +)	0	0	0	0		0	0	5	0	0	5	100% (5/5)	100% (5/5)
Total	18	6	6	30		90% (27/30)	80% (24/30)	30	0	0	30	86.67% (26/30)	83.33% (25/30)

*Abbreviations:* Nx: Number of treatments

### The outcome of CR and PR in 5-ALA PDT and LEEP group

From Table 4, in 5-ALA group, among 23 CIN1 patients, 69.57% (16/23) achieved CR, while CR was 83.33% (5/6) among 6 CIN2 patients and 100% (1/1) in 1 CIN3 patients, moreover, there was one patient degraded from CIN2 to CIN1 with PR was 16.67% (1/6). In LEEP group, CR was 71.43% (10/14), 88.89% (8/9), 100% (2/2) in CIN1, 2, 3 patients, respectively. Moreover, there was also one CIN2 patient degraded to CIN1 with PR 11.11% (1/9). Among different CIN group, there was no statistic difference between 5-ALA PDT and LEEP.

**Table 4:** The comparation of CR, PR in CIN1-3 patients (*n* = 55)

	Outcome	5-ALA PDT	LEEP		P
CIN1	CR	69.57% (16/23)	71.43% (10/14)		0.904
	PR	0% (0/23)		0% (0/14)	/
CIN2	CR	83.33% (5/6)		88.89% (8/9)	1.000
	PR	16.67% (1/6)		11.11% (1/9)	1.000
CIN3	CR	100% (1/1)		100% (2/2)	/
	PR	0% (0/1)		0% (0/2)	/

*Abbreviations:* CR: Complete remission; PR: Partial remission

In total, in terms of 5-ALA group, CR was 73.33% (22/30), PR was 3.33% (1/30), 5 patients remained ASCUS after the treatment and 2 CIN1 patients showed no response to 5-ALA PDT (Table 2). In LEEP group, 5 patients were only infected by HPV, CR was 84.00% (21/25), PR was 4.00% (1/25) and 4 CIN1 patients left was insensitive to LEEP (Table 2). Compare the CR and PR of these two groups, there was no significant difference (Table 5).

**Table 5:** The comparation of CR, PR and HPV remission (*n* = 60)

	CR		PR		HPV remission	
	n	rate%	n	rate%	n	rate%
5-ALA PDT	22(30)	73.33%	1(30)	3.33%	16(27)	59.26%
LEEP	21(25)	84.00%	1(25)	4.00%	14(26)	53.85%
P	0.340 > 0.05		1.000 > 0.05		0.691 > 0.05	

*Abbreviations:* CR: Complete remission; PR: Partial remission

### The HPV remission rate in 5-ALA PDT and LEEP group

From Table 3, in 5-ALA PDT group, among 23 CIN1 patients, 91.30% (20/23) patients had HPV infection and 78.26% (17/23) had HR-HPV infection. In terms of 6 CIN2 patients and 1 CIN3 patients, all got HPV and HR-HPV infection. As for LEEP group, 93.33% (14/15) CIN1 patients had HR-HPV infection, 66.67% (6/9) CIN2 had HPV and 55.56% (5/9) CIN2 had HR-HPV infection. 2 CIN3 patients both were infected by HR-HPV. In total, 27 of 30 (90.00%) patients in 5-ALA PDT group and 26 of 30 (86.67%) patients in LEEP group got HPV infection. The above illustrated that not all patients with CIN were infected by HPV. The HPV remission rate was 59.26% (16/27) in 5-ALA group and 53.85% (14/26) in LEEP group. There was also no statistical significance of HPV remission rate between these two groups (Table 5).

### The side effects of 5-ALA PDT and LEEP

During the period of 5-ALA PDT, the adverse effects were minimal. Patients’ complaints focused mainly on the pain such as slight dysmenorrhea in the lower abdomen during the treatment, and temporary increased vaginal secretions, which would be relieved 1-week after the treatment. Minor bleeding, pain, and infection after the first 24 h after the treatment were the main side effects of LEEP, however, which also could be avoided by proper nursing.

## Discussion

LEEP is the common methods for treating CIN, however, LEEP has presented its drawbacks, one of the most serious side effects is the risk of miscarriage [6], which leads many young women with CIN to be reluctant to choose LEEP, moreover, LEEP does not recognize the lesion, sometimes leading to the positive margin and a second surgery. 5-ALA PDT has been verified as effective in treating CIN by increasing number of studies in recent years [11–14]. However, to date, there is no relevant studies about comparison between LEEP and 5-ALA PDT. In our study, we retrieved medical records of patients who received LEEP and 5-ALA PDT at the department of obstetrics and gynecology and laser medicine in Chinese PLA General Hospital from 2012 to 2019. To make them comparable, PSM was applied and the match tolerance was set as 0.03 and finally 30 couples (60 patients) were paired.

Among 30 patients treated with LEEP, there were 14 patients diagnosed as CIN1 and 5 patients only infected with HPV. LEEP is the first line treatment for CIN2 and 3 not for CIN1 according to the WHO Guidelines for Screening and Treatment of Precancerous Lesions for Cervical Cancer Prevention (2014). While in LEEP group, 93.33% (14/15) CIN1 patients had HR-HPV infection for at least 2 years. According to Gynecologic oncology handbook: an evidence-based clinical guide [15], in the case of CIN1 patients with delayed HPV infection for more than two years, or ineffective conservative treatment, surgery like LEEP can be performed, which has been reported in many clinical researches [16–19]. In addition, in our research, the mean age of the CIN1 patients in LEEP group was 37 and only 2 patients were under 30, most of whom had no desire for fertility and tend to choose LEEP. Moreover, bothered by HPV infection, some CIN1 patients insisted on further treatment such as LEEP. For the patients only with HPV ( +) included in our research, the age of them was 46, 28, 44, 41, and 51, respectively (the detailed data were presented in Table 2), and they have each already given birth to at least one child. Moreover, the antiviral treatment has been ineffective for a long time (more than 5 years). The patients worried about the deterioration of the disease, and has no need for fertility, hence, they chose to receive LEEP.

By comparing the effectiveness, 5-ALA PDT showed the same ability of treating different CIN group as LEEP. The CR of 5-ALA PDT was 73.33% (22/30) comparing to 84.00% (21/25) of LEEP. One meta-analysis included 4 RCTs which reported that 77 of 120 (64.2%) patients in PDT group achieved primary complete remission at the end of the 3-month follow-up period [20], which was consistent with our result. Moreover, among 30 patients of 5-ALA PDT, 5 patients were diagnosed as ASCUS when they quitted treatment. We defined ASCUS as invalid as ASCUS has the potential to progress into CIN or cervical cancer if there is no intervention and proper management [21]. It is important to note that ASCUS (atypical squamous cells of undetermined significance) is considered less severe than ASC-H (atypical squamous cells, cannot exclude high-grade squamous intraepithelial lesion). ASC-H indicates a higher suspicion of high-grade squamous intraepithelial lesion. Regular inspections and follow-up are recommended to monitor and manage ASC-H cases appropriately. Moreover, the results would be desirable if the patients with ASCUS adopted the repeated treatment. Hence, 5-ALA PDT has the comparable results as LEEP. Beyond ASCUS, there was 2 patients and 4 patients in 5-ALA PDT and LEEP group showed resistance to the therapy, respectively, which illustrated there was no perfect treatment for all kinds of patients and a new modality for CIN should be invented.

In terms of different grades of CIN, 5-ALA PDT showed same efficacy as LEEP (Table 4). CR was 69.57% (16/23) in 5-ALA PDT group comparing to 71.43% (10/14) of LEEP group when it comes to CIN1. Similarly, among CIN2 patients, CR and PR in 5-ALA PDT and LEEP group showed no statistic difference. Moreover, there was only 3 CIN3 patients included in our study (1 for 5-ALA PDT and 2 for LEEP). Although the efficacy of topical PDT has been verified in treating CIN3, [22] due to the lack of large-scale clinical trials of 5-ALA PDT treating CIN3 patients, the number of CIN3 patients treated by local 5-ALA PDT was only one in our study. Hence, the samples were too small to compare 5-ALA PDT and LEEP. However, in the future, we will conduct the study to demonstrate the clinical effectiveness of 5-ALA PDT for treating CIN3 and further compare 5-ALA PDT and LEEP.

There was no statistical significance about HPV remission rate, 5-ALA PDT was 59.26% and LEEP was 53.85%. Moreover, positive outcomes have also been reported 5-ALA PDT’s efficacy for an antiviral effect when it has been used for HPV-related diseases, such as condylomata [23, 24] and epidermodysplasia verruciformis [25, 26]. We also found not all CIN patients were infected by HPV, which illustrated continuous HPV infection contributes to CIN, while not all CIN are attributed from HPV infection, however, this argument should be further investigated due to only 18 HPV test kits were used in our study.

In our study, there were 12 patients received more than one time PDT, which means PDT is a repeatable and safe procedure. However, during the LEEP procedure, it is crucial for doctors to carefully balance the objective of completely excising the entire lesion while minimizing the removal of healthy cervical tissue [27]. The most crucial risk factor for recurrence of CIN is incomplete removal of the lesion [28], but PDT can solve the problem, if the HPV genotyping or cytological tests after the treatment suggest the undesirable results, the repeated PDT can be adapted. Moreover, the topical application of a photosensitizer provides a significant advantage by resulting in reduced systemic exposure and rapid clearance without cutaneous photosensitivity.

Even though 5-ALA PDT showed much potential on treating CIN, LEEP was still the standard treatment. LEEP can have postoperative pathological results for the judgement of whether all the lesion were removed and PDT cannot have, however, postoperative pathology may result in secondary damage. The effectiveness of ALA-PDT needs multicenter, large sample size clinical trials. Due to constraints in time and resources, this study has its limitations. As CIN is a precancerous condition that may evolve over time, our assessment is restricted to cytological tests and HPV genotyping within 3–6 months post-treatment. The absence of longer-term follow-up data hinders a comprehensive exploration of the prolonged postoperative intervention effects.

To sum up, even though the number was limited to some extent, these patients were representative for comparing. In our study, topical 5-ALA PDT has been verified as a non-surgical tissue-preserving treatment for CIN and persistent HPV infection compared to LEEP. In the future, more prospective studies will be required to provide more potent evidence.

## Conclusion

Topical 5-ALA PDT for cervical lesions seems to be a non-invasive, repeatable procedure with minimal side effects, preserving cervical function which can be easily performed on outpatient basis. In contrast to LEEP, PDT causes no substantial cervical stroma destruction thus reducing the risk of a possible subsequent incomplete cervix. Moreover, in most cases, PDT with 5-ALA is a successful treatment with comparable results to LEEP.

## Data Availability

The data that support the findings of this study are available from the corresponding author [Ying Gu guyinglaser301@163.com] upon reasonable request.
